# American Society of Clinical Oncology’s Global Oncology Leadership Task Force: Findings and Actions

**DOI:** 10.1200/JGO.17.00060

**Published:** 2017-08-11

**Authors:** Gabriel N. Hortobagyi, Doug Pyle, Eduardo L. Cazap, Nagi S. El Saghir, Lawrence N. Shulman, Gary H. Lyman, Lowell E. Schnipper, Clement Adebayo Adebamowo, David R. Gandara, Julie Vose, Sandra L. Wong, Peter Yu

**Affiliations:** **Gabriel N. Hortobagyi**, The University of Texas MD Anderson Cancer Center, Houston, TX; **Doug Pyle**, American Society of Clinical Oncology, Alexandria, VA; **Eduardo L. Cazap**, Sociedad Latinoamericana y del Caribe de Oncología Médica, Buenos Aires, Argentina; **Nagi S. El Saghir**, American University of Beirut Medical Center, Beirut, Lebanon; **Lawrence N. Shulman**, University of Pennsylvania, Philadelphia, PA; **Gary H. Lyman**, Fred Hutchinson Cancer Research Center, Seattle, WA; **Lowell E. Schnipper**, Beth Israel Deaconess Medical Center, Boston, MA; **Clement Adebayo Adebamowo**, Institute of Human Virology, Baltimore, MD; **David R. Gandara**, University of California Davis Comprehensive Cancer Center, Sacramento, CA; **Julie Vose**, University of Nebraska Medical Center, Omaha, NE; **Sandra L. Wong**, Dartmouth-Hitchcock Medical Center, Lebanon, NH; and **Peter Yu**, Hartford HealthCare, Hartford, CT.

## Abstract

In response to rising cancer incidence and mortality rates in low- and middle-income countries and the increasingly global profile of ASCO’s membership, the ASCO Board of Directors appointed the Global Oncology Leadership Task Force (Task Force) to provide recommendations on ASCO’s engagement in global oncology. To accomplish its work, the Task Force convened meetings of global oncology experts, conducted focus group discussions with member groups, did site visits to South America and India, and met regularly to analyze the findings and develop recommendations. Task Force findings included global concerns, such as access to care, and specific concerns of middle- and low-resource settings. The need to strengthen health systems and the importance of alliances with a range of international cancer stakeholders were emphasized. Task Force recommendations to the ASCO Board of Directors were based on a three-part global oncology strategy of professional development, improvement of access to quality care, and acceleration of global oncology research. Specific areas of focus within each of these strategic pillars are provided along with an update on areas of ASCO activity as these recommendations are implemented.

## INTRODUCTION

Cancer incidence and mortality in low- and middle-income countries (LMCs) have been rising steadily over the past several decades. In 2012, the International Agency for Research on Cancer (IARC) estimated that approximately two thirds of all cancer deaths and nearly 60% of new cancer cases occur in LMCs. Furthermore, IARC has projected that by 2030, new cases of cancer in LMCs will be nearly double those in high-income countries, and more than twice as many cancer deaths will occur in LMCs than in high-income countries.^1^ Cancer has become more broadly recognized by the global community as a global health priority, as evidenced by the historic 2011 United Nations High Level Meeting on Non-Communicable Diseases.^2^

These epidemiologic trends are reflected in the increasingly global profile of ASCO’s membership: Approximately one third of ASCO members practice outside the United States, and of these international members, one quarter practice in LMCs, which represents a significant and growing constituency. Thus, the ASCO Board of Directors has made it a priority to consider their needs and interests.

The Global Oncology Leadership Task Force (Task Force) was formed by the ASCO Board of Directors to provide recommendations on ASCO’s engagement in global oncology. More specifically, the Task Force was charged with identifying ASCO programs and services that have the potential to address unmet needs in oncology communities outside the United States as well as other cancer-related issues that the international cancer community is not fully addressing. The Task Force collaborated with ASCO’s International Affairs Committee and other ASCO committees to evaluate ASCO resources and opportunities to leverage other relevant components of ASCO toward global goals. The Task Force was chaired by Gabriel Hortobagyi, MD, and staffed by Doug Pyle, Vice President for International Affairs.

## METHODS

To accomplish its work, the Task Force performed the following activities from July 2014 to March 2016:

1. An initial conference call to collect input on the Task Force’s agenda;
2. Quarterly conference calls that focused on particular issues or themes on the Task Force agenda;
3. Focus group discussion with past recipients of the International Development and Education Award (IDEA) during the 2015 ASCO Annual Meeting;
4. Participation in a workshop organized by ASCO and the College of American Pathologists (CAP) in July 2015 to identify strategies for enhancing the pathology workforce in LMCs;
5. Visits to Argentina, Brazil, and Uruguay in March 2015 and India in September 2015 by ASCO Past President Peter Yu, MD, and ASCO Vice President for International Affairs Doug Pyle to gather insights into challenges and opportunities in these settings;
6. Hosting of a Global Oncology Summit for academic, corporate, and government global oncology leaders in January 2016.

The Task Force wrote a white paper that outlined its finding and recommendations, which was presented to the ASCO Board of Directors in June 2016.

## RESULTS

### Global Oncology: Global Concerns

Although definitions vary, global health has been described as an “area for study, research, and practice that places a priority on improving health and achieving health equity for all people worldwide.”^3^ Global oncology is a more recent term that generally refers to the application of the concepts of global health to cancer and implies an approach to the practice of oncology that acknowledges the reality of limited resources in most parts of the world.

Although its mandate covered a wide range of issues, geographies, and practice settings, the Task Force identified common themes and concerns among the diverse stakeholders consulted. These themes included the following:

- Professional development: support for the highest quality of specialty training, continuing professional education, and career development in global oncology. This includes:
  ∘ The need to train allied health care personnel (eg, nurses, community health workers) in aspects of oncology care to leverage the existing health care workforce, particularly where the specialist workforce is severely limited;
  ∘ The need for optimal training and postgraduate education of oncology specialists worldwide;
  ∘ Recognition in high-income countries of global oncology as a field for formal professional development and as a legitimate, research-oriented academic field.
- Quality of care:
  ∘ Access to care: while the affordability of care relative to available resources varies, concern about the rising cost of care is certainly a global one.
  ∘ Quality standards and patient-centered outcomes.
  ∘ Cultural barriers to quality improvement such as resistance to the use of narcotics for pain control or a public perception that cancer is an untreatable disease.^4^
- Research: shared interest in research and concerns about regulatory and funding challenges associated with cancer research.

These common concerns offer opportunities for collaborative efforts around the world to develop optimal solutions to these challenges, which in all cases should be context-specific, taking into account the local environment, resources available, financial considerations, and other factors. Global oncology requires a paradigm shift from a model of taking knowledge generated in a developed location and disseminating it to a less-developed location to a multipolar model where solutions are generated and shared across multiple settings. Indeed, during the Global Oncology Summit, reverse innovation was highlighted as an opportunity to accelerate discovery by researching innovative approaches pursued in LCMs that could be globally applicable.

### Resource Stratification: Middle Resource and Low Resource

The Task Force heard significant commonality in terms of issues and concerns, and deliberations also highlighted significant distinctions among practice settings that were driven in part by the varying resources available. In these discussions, countries typically are categorized as high income, middle income, and low income. On a national economy basis, the World Bank defines middle income economies as those with a gross national income (GNI) per capita of > $1,045 but < $12,736, low income as a GNI per capita of ≤ $1,045, and high income a GNI per capita of ≥ $12,736.^5^ The stratification of cancer control interventions by resource availability was pioneered by the Breast Health Global Initiative.^6^

Although useful as a framework, within countries categorized as middle income (eg, India, Brazil), low-resource rural settings can coexist with urban areas with highly advanced facilities. Furthermore, a country may have a middle-resource economy but have a high resource health system or a high resource economy and an underdeveloped health system.

#### Middle-resource countries.

To better understand middle-resource country (MRC) issues and opportunities in depth, intensive visits by ASCO representatives to Brazil, Argentina, and Uruguay (March 2015) and India (September 2015) occurred under the auspices of the Task Force. MRCs were also a theme of the Global Oncology Summit at ASCO’s headquarters in Alexandria, VA, in January 2016. Generally, MRCs appear to offer an infrastructure conducive to ASCO having a significant impact, including established systems for medical education (eg, oncology specialty training) and health care delivery systems and relatively stable and growing economies with a growing middle class that places a priority on health, political stability, and governance.

The visits to the MRCs revealed an overall high credibility level for ASCO and its meetings, guidelines, and products. The interest in standards may be partly due to variability in oncology training and professional certification in some MRCs (eg, at the time of ASCO’s visit in 2015, oncology training in Argentina could be obtained through a 5-year residency or through a shorter university course certification, but more recently, the Argentine Ministry of Health reportedly initiated a harmonization effort with respect to oncology training) and an interest among oncology training programs to establish quality standards and differentiate their programs on the basis of quality. ASCO and the European Society for Medical Oncology actively support medical oncology training worldwide and recently released updated recommendations for medical oncology training.^7^

Similarly, the Task Force perceived in MRCs a growing and dynamic private hospital sector that sees quality and certification as market differentiators. For example, private hospitals in India promote their adherence to the Joint Commission International certification. ASCO’s Quality Oncology Practice Initiative (QOPI) and, even more so, QOPI Certification, offer an opportunity for ASCO to respond to this organic, market-driven interest in quality standards. ASCO also heard top-down interest in programs like QOPI from government authorities in support of ministries of health efforts to improve health care quality and reduce care disparities.

Representative of nearly 40% of the world’s population and > 20% of its cancer population (on the basis of 1-year prevalence data^8^), India and China are particularly critical to global cancer control efforts. Both countries have a rising middle class with growing expectations of the services (particularly health care services) it receives. These countries represent new markets for health insurance and health care delivery and have a growing cancer burden and an urgent need to develop integrated health systems that effectively respond to this burden.

Finally, many barriers to research exist in MRCs. Discussions with researchers in Argentina, Brazil, and India in particular highlighted gaps (in terms of funding and infrastructure) that prevent promising basic research from resulting in productive translational research. Challenges also include an environment that does not support academic research, including lack of protected time, limited training in the design and conduct of research, and lack of the requisite trained research support staff. The National Cancer Institute (NCI) Center for Global Health (CGH) is actively engaged in this issue and has efforts under way to encourage national governments to support noncommercial trials in their countries and to translate research results into practice. The CGH currently offers a portfolio of programs to support research training (as does ASCO) and has launched a Latin American Cancer Research Network as part of this strategy.

#### Low-resource countries.

In low-resource countries (LRCs) the barriers to quality cancer care are typically more numerous and fundamental than in MRCs. The Task Force identified five themes:

1. Human resource limitations in terms of training (lack of knowledge about cancer in general and cancer care specifically): Many countries have few or no trained oncologists, so overcoming this knowledge gap is particularly challenging. In addition a limited number of physicians and clinical staff frequently exist. The general population receives care at government-run hospitals where insufficient staffing results in delays in diagnosis and treatment and during which time the cancer advances. Efforts in cancer prevention are limited.
2. Limitations in key facilities and capabilities, for example, the lack of pathology capacity to determine accurate cancer diagnoses: The lack of pathology or cytology delays treatment, results in incorrect treatment, or makes certain treatments not possible. In addition, surgical and radiation oncology capabilities and cancer registries and associated national cancer control plans are frequent limitations.
3. Limited access to medicines: Both generic and novel drugs either are not available or are available but are too expensive for the public system to cover or for patients to pay out of pocket. Pain medications in particular are low-cost agents that could have a major impact on the quality of life of patients with cancer in these settings.
4. Geographic challenges: Often, treatment facilities are distant or centrally located, and the transportation infrastructure is limited. As a result, chemotherapy regimens or radiotherapy that requires frequent visits sometimes are not performed.
5. Importance of engaging ministries of finance to increase the allocation of resources to health care.

Despite these challenges and others, models exist that prove effective in improving cancer care delivery and patient outcomes. Several academic cancer centers in the United States are implementing robust, multifaceted, capacity-building programs with collaborators in LRCs. The Dana-Farber/Partners In Health program in Rwanda is one model.^9^ Some critical success factors cited include the following (L.N. Shulman, personal communication, July 7, 2015):

- Intermittent training is essential but on its own, is not adequate and must be accompanied by ongoing engagement and support focused on implementation of care systems and sustainability.
- Training by visiting oncologists includes not only lectures but also inpatient ward rounding with teaching in the trenches.
- Cancer care infrastructure must include high-quality and timely pathology; pharmacy support; skilled oncology nursing support; and, ideally, a database to track and follow patients and to evaluate the safety and efficacy of treatment programs (additional core infrastructure requirements [eg, surgical capabilities] were also discussed).
- Specific and parallel nurse training is essential.
- Detailed disease-based written pathways of care from diagnostics to treatment to follow-up are essential and must be accessible and usable.
- Training and written disease-based pathways must be context specific.

The fundamental needs in LRC practice settings and the critical lack of funding resources to support the delivery of basic care in these settings have led to a growing group of cancer leaders, including the Union for International Cancer Control (UICC) Past President and Task Force member Eduardo Cazap, MD, PhD, and UICC past president Franco Cavalli, MD, to propose a global fund for cancer similar to the Global Fund to Fight AIDS, Tuberculosis and Malaria. Given limited resources, opportunities for advancing cancer prevention in these settings are especially critical, including for campaigns against the use of tobacco products, betel nuts, and other carcinogens; education about the long-term benefits of vaccines against hepatitis viruses and human papillomavirus; and identification and management of *Helicobacter pylori* infection.

### Health Systems Strengthening

The Task Force discussed the need for ASCO programs to be integrated within existing health care systems of the countries in which they are being implemented. Such an approach makes ASCO programs more relevant and adaptable to address the realities of that health care setting, ensures that the program will have a sustainable impact that is aligned with other efforts to improve the health care system, and considers critical components of the cancer care delivery system on which oncology depends.

The collaboration between ASCO and CAP is an example of a systemic approach to programming. Under this collaboration, ASCO and CAP are developing tools and resources to assess pathology capacity and address pathology gaps in four pilot countries (Haiti, Honduras, Uganda, and Vietnam) with an aim to improve pathology capacity in LMCs. In proposing this collaboration to CAP, ASCO perceived a critical deficiency in the cancer care delivery system, and through an alliance is working with partners to address that deficiency. With the assumption that this collaboration will be successful, ASCO could consider a collaboration with sister societies to address other gaps in cancer care delivery in LMCs.

The integration of programs within an existing health care system also means adaptation to and leverage of available resources. In LRCs and even MRCs, a critical lack of formally trained medical oncologists and other oncology specialists exists, which necessitates the consideration of an unconventional oncology workforce to enhance overall cancer care capacity. In middle-resource settings, organ specialists (eg, pulmonologists who treat lung cancer) are key constituents. In low-resource settings, health care delivery is focused on a primary care workforce. The Dana-Farber experience with training primary care providers in Rwanda to deliver cancer treatment has already been mentioned. In India, a similar program internally initiated by an ASCO member provides generalist training to reach outlying communities. The scarcity of trained nurses in most LMCs also is a key component of the health care workforce shortage in these settings, and an opportunity to work with the Oncology Nursing Society (ONS) was identified.

Finally, the Task Force discussed the value of integrating the range of programs and products in ASCO’s portfolio on a national level. Although various ASCO international programs commonly are linked in a particular country, this process remains relatively informal. For example, past IDEA recipients often organize ASCO international courses in their countries and apply for innovation grants. The Task Force suggests that ASCO could achieve more through a country-focused approach that engages national governments and health systems more formally and that more consciously draws on ASCO offerings in a planned manner.

### Alliances

A consensus existed among Task Force members about the importance of strategic alliances in supporting and extending ASCO’s international efforts. ASCO collaborates with a strong network of oncology societies to organize programs around the world, and these will continue to grow. In addition, the Task Force identified seven other alliances that could be further developed or initiated:

1. International agencies such as the WHO, IARC, and other United Nations bodies: Work with these agencies will align ASCO’s international programs with broader international initiatives and amplify their impact. The successful collaboration among ASCO, UICC, and WHO to add cancer medicines to the WHO Essential Medicines List is an example of work that can have a global impact on cancer care.
2. NCI and other US government agencies: While the NCI CGH and ASCO have collaborated on training courses, the IDEA program, and other areas, ASCO and NCI could deepen this collaboration in such areas as development of research programs and research funding for global oncology. In addition, ASCO has the opportunity to develop relationships with other government agencies that may view its international programs as supportive of health diplomacy and that may be potential funding sources for ASCO’s international programs (eg, the US Agency for International Development).
3. Foreign national governments: As detailed previously, experiences in South America and India where ASCO members have brokered meetings between ASCO representatives and ministers of health present a model for ASCO to develop a relationship with national governments, promote anticancer policies, and develop programmatic collaborations with health ministries and other government agencies.
4. Other US medical associations: ASCO’s collaboration with CAP to promote pathology capacity in LMCs is a potential model for ASCO to work more substantially with other associations in domains of need. For example, ASCO and ONS could collaborate more to support nursing capacity in LMCs, and ASCO and the International Association for the Study of Lung Cancer could work more closely given the latter’s international network in lung cancer. Possibilities for collaboration between ASCO and the Infectious Diseases Society of America were suggested during the Global Oncology Summit given the expertise of the infectious diseases community in conducting large-scale international programs and the relative frequency of infection-related cancers in low-resource settings.
5. US academic cancer centers that conduct global oncology programs: The Global Oncology Summit, which had a large representation from these academic centers, highlighted the potential synergies between ASCO and these centers. One was in the development of global oncology as an academic field, and many representatives from these centers expressed a need for the field to be more formally recognized, research to be better funded, and barriers to fellows who pursue an interest in global oncology to be addressed (including the provision of protected time for faculty to engage in global oncology activities) and saw a role for ASCO to align these needs with its other professional development activities. Another synergy was in collaborations where academic centers perform intensive programs in LMCs (eg, Rwanda, Kenya). ASCO could collaborate with, learn from, and help to disseminate promising models from these initiatives.
6. Public advocacy organizations: ASCO has the opportunity to enhance its role internationally through partnerships and its international members to raise public awareness about cancer as a disease, its potential curability, and the importance of early diagnosis and timely and effective treatment. This could be expanded to include education of the local media and political decision makers.
7. Individual oncology leaders in the countries. Although substantial gains have been made through the IDEA program and, more recently, international member participation in the Leadership Development Program, there is an opportunity to expand ASCO’s leadership development capabilities and experience internationally, possibly through local leadership training in conjunction with national society partners. These leaders would be critical for engaging their own governments and professional societies in advancing quality care.

## PUTTING THE FINDINGS INTO ACTION

ASCO offers a robust portfolio of international programs^10^ that consists of three mutually supportive pillars: professional development, quality improvement and research. Each of these domains represents an area of ASCO strength.

1. Professional development cultivates current and future oncology practitioners and leaders who serve as change agents to advance global oncology. Examples of existing ASCO programs are the IDEA program, Leadership Development Program, Virtual Mentors program, and ASCO/European Society of Medical Oncologists Global Curriculum.
2. Quality improvement programs and tools engage these leaders, members, and other stakeholders to drive improvements in cancer care delivery around the world. Existing ASCO programs in this domain are international training courses, the International Cancer Corps program, QOPI, and resource-stratified guidelines.
3. Research (training, funding, and dissemination) can, in turn, inform quality improvement strategies and provide a pathway for professional development. Examples of existing ASCO international programs are International Innovation Grants, *Journal of Global Oncology*, and International Clinical Trials Workshops.

Each pillar requires active advocacy on the part of ASCO to key audiences (including the education of governments and potentially the public) and strong alliances with key stakeholders. Through this framework, ASCO is now pursuing a number of new initiatives to put the findings of the Task Force into action.

### Professional Development

#### Promoting the recognition of global oncology as an academic field.

ASCO will be engaging various stakeholders to support the transition of global oncology from an informal field of largely voluntary activity to a formal field with a strong research component and recognized value of oncology training and the practice of oncology. Such an initiative builds on ASCO’s expertise that supports the professional development of domestic and international members, and ASCO’s recent efforts to formalize global oncology through *Journal of Global Oncology*, the global oncology track at the ASCO Annual Meeting, and the Global Oncology Symposium.

#### Training of nonspecialists in oncology principles.

Recognizing that the demand for oncology services will exceed the supply of specialists in many LMCs for the foreseeable future, ASCO is reviewing models for the training of nonspecialists in oncology principles and cancers commonly found in their region or communities. ASCO can learn from approaches currently pursued in Rwanda, India, and Canada, and can build on its existing Cancer Control for Primary Care course to educate health care workers in underserved communities.

### Quality Improvement

#### Accelerating QOPI Certification internationally.

QOPI Certification has a significant potential to promote a global standard of excellence in cancer care and to motivate cancer practices to improve the care delivered. In 2016, ASCO awarded QOPI Certification to its first practice outside the United States: the Contemporary Oncology Team practice in Athens, Greece. Earlier this year, certification was awarded to a practice in Brazil, and based on this experience, ASCO will promote QOPI Certification in other countries.

#### Supporting improved cancer control in LMC cities.

As new investments in the cancer care infrastructure are made in middle-resource (and some low-resource) settings, ASCO can offer trusted, scientific guidance on the essential elements for an effective quality cancer center and cancer care delivery. ASCO is proud to be a founding collaborator with UICC on the City Cancer Challenge launched in January 2017. With 54% of the world’s population already living in cities, which is expected to rise above 66% in the coming decades, C/Can 2025: City Cancer Challenge will address the urgent need for fully functional, comprehensive cancer solutions in urban areas to reach the majority of the world’s population. The first three cities who have committed to the challenge are Asunción in Paraguay, Cali in Colombia, and Yangon in Myanmar. These key learning cities will provide insights on how the international community, local civil society, and public sector can best work together. ASCO and its member volunteers will be contributing technical assistance to the program and linking the program requirements with relevant ASCO programs and resources.

#### Extending ASCO international programming beyond oncology.

Through an alliance with CAP, ASCO is extending its programs in LMCs to include pathology capacity development, which links CAP pathology expertise and tools with ASCO’s international network and programmatic expertise. This alliance with CAP can be a model for ASCO to collaborate with other organizations in other cancer control domains (IARC in registries, ONS in oncology nursing).

### Research

#### Making global oncology a formal component of the ASCO Annual Meeting.

The ASCO Annual Meeting serves as a platform to highlight global oncology issues and research, to promote a dialog about these topics from different perspectives, and to raise awareness of these issues among attendees. This has been realized through the Global Oncology Symposium that was organized with the 2015 and 2016 ASCO Annual Meetings, and now the Global Health Track that was started with the 2017 Annual Meeting.

#### Creating Conquer Cancer Foundation research awards for global oncology.

ASCO’s philanthropic affiliate the Conquer Cancer Foundation (CCF) offers a grants and awards program that includes the Young Investigator Awards; Career Development Awards; and for research in LMCs specifically, International Innovation Grants. In response to the need and interest in global oncology research that the Task Force identified, the CCF is now developing new research awards to support formal and robust global oncology research. The CCF Global Oncology Grants Task Force has defined award criteria and terms and announced the first awards at the 2017 ASCO Annual Meeting.

#### Deepening of CCF involvement in global oncology.

As ASCO’s philanthropic affiliate, CCF can further raise awareness of the critical need for additional resources for global oncology interventions and the impact of these interventions on patient care around the world. The global oncology mission may resonate with new donor sources, and high-profile forums, such as the World Economic Forum meeting in Davos, Switzerland, may raise public and philanthropic awareness of international cancer issues. In fact, the UICC has established a productive collaboration with the World Economic Forum and launched the City Cancer Challenge at Davos this year.

In conclusion, by publishing the findings of the Task Force and reporting some of the follow-up actions, we hope to inform the dynamic discussions of today on the alarming challenges in global oncology and the ways to address these challenges. The planned actions by ASCO as outlined in this article are intended to address some of these challenges, but ultimately, a suitable response will require many actions by many organizations in all sectors of our global community. The leadership and membership of ASCO look forward to this collaboration and to realizing the improved patient outcomes that we all envision.
